# 948. Ventricular Assist Device Infection due to *Non-tuberculous Mycobacteria* Leading to Successful Heart Transplantation: A Case Report and Literature Review

**DOI:** 10.1093/ofid/ofab466.1143

**Published:** 2021-12-04

**Authors:** Carlos s Saldana, Susan J Rehm, Susan J Rehm, Christine E Koval, Shinya Unai, Zhen-Yu (Michael) Tong

**Affiliations:** 1 Emory University School of Medicine, Atlanta, GA; 2 Cleveland Clinic, Cleveland, OH; 3 Cleveland Clinic Foundation, Cleveland, OH

## Abstract

**Background:**

A 59-year-old man with acute myelogenous leukemia and matched allogeneic hematopoietic stem-cell transplant and non-ischemic cardiomyopathy requiring a left ventricular assist device (LVAD; HeartMate II™) was admitted after the spontaneous rupture of an abdominal fistula in his lower abdomen, and cloudy discharge from the driveline (DL) exit site for months. Figure 1 Denied systemic symptoms but had leukocytosis. CT scan of the abdomen revealed soft tissue enhancement around the LVAD DL Figure 2. Cultures from DL discharge grew *Mycobacterium chelonae*. He underwent incision and drainage (I&D) of the abdominal fistula, with unroofing of the tissue over the DL. Antimicrobial course is summarized in Table. He was thought to be an appropriate heart transplant candidate one month later. Cultures from the LVAD sites were negative. He completed antimicrobials for 10 weeks after transplant. 32 months after heart transplant he has no signs of *M. chelonae* infection.

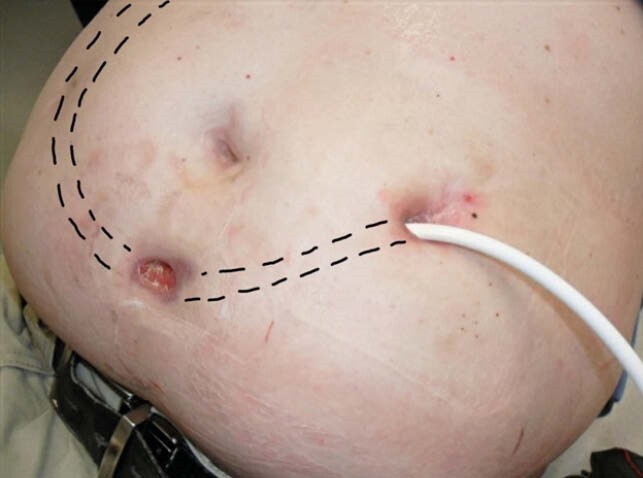

Figure 1. Driveline exit site with scant cloudy discharge and a shallow 3 x 2-centimeter open lesion inferior to the umbilicus. Driveline track in dotted line.

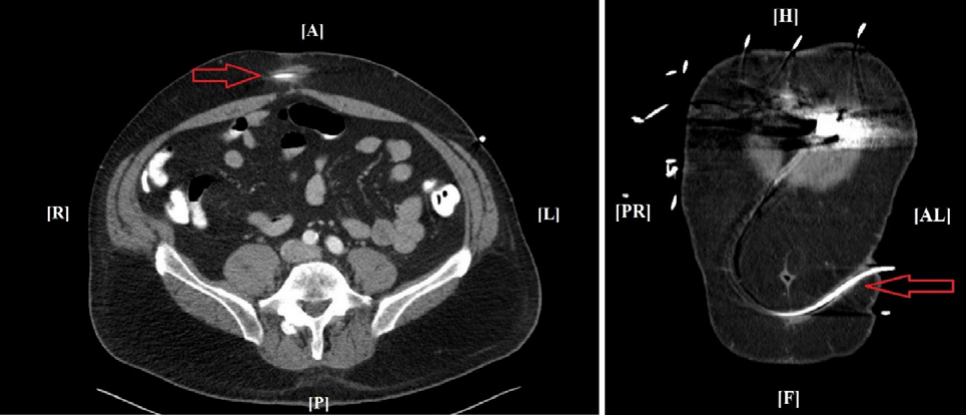

Figure 2. CT scan of the abdomen revealed soft tissue enhancement (red arrows) around the LVAD driveline in the lower abdomen.

**Methods:**

We performed a literature review of all published cases involving *Non-Tuberculous Mycobacteria* (NTM) and LVADs. Collected: date, sex and age, onset, organism, type of LVAD, transplant, surgical debridement, antimicrobials, outcome.

**Results:**

A total of 11 patients with LVAD infection due to NTM have been described in the literature. Four cases of NTM LVAD infection culminated in heart transplantation. Cases are summarized in Table 1. All transplanted cases had an indolent presentation and driveline discharge, without systemic symptoms. All underwent I&D and/or de-roofing of the driveline and were treated with at least two active antimicrobials for an extended course ranging from 4 months and up to 17 months after transplant. All cases did well from the infection standpoint. One died within 12 months from transplant rejection.

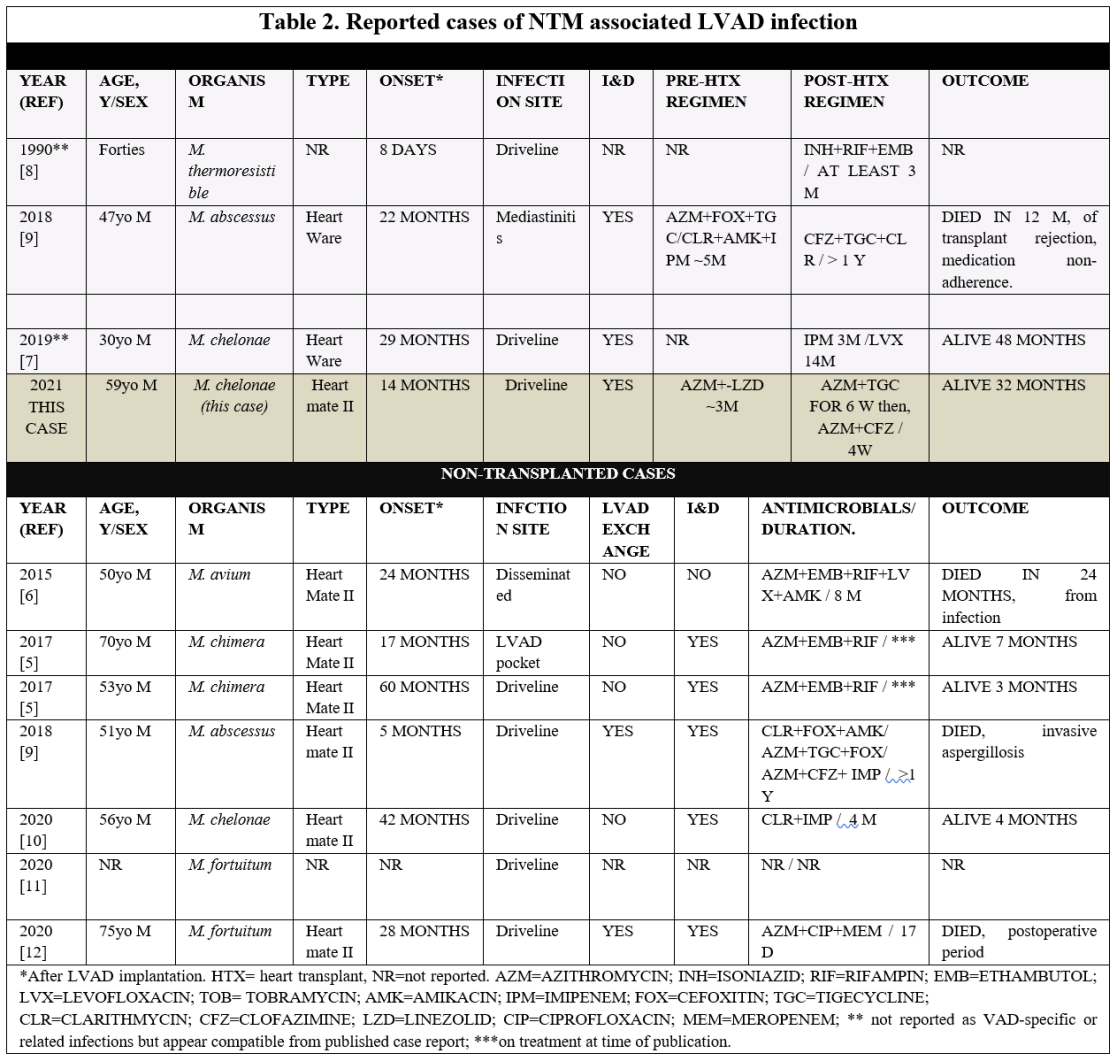

**Conclusion:**

Treatment with a combination with at least two active agents is recommended and continued for many months. Effective surgical debridement of affected tissue and unroofing of the driveline beyond the affected velour, along with the removal of the infected device at the time of cardiac transplant, is key to success.

**Disclosures:**

**Susan J. Rehm, MD**, Lilly (Individual(s) Involved: Self): Shareholder; Merck (Individual(s) Involved: Self): Shareholder; Pfizer (Individual(s) Involved: Self): Shareholder

